# Severe acute intestinal graft versus host disease requiring surgical resection

**DOI:** 10.1002/jha2.24

**Published:** 2020-06-03

**Authors:** Hisashi Ishida, Takuo Noda, Seiji Kawano, Maho Sato, Hirokazu Tsukahara, Akira Shimada

**Affiliations:** ^1^ Department of Paediatrics Okayama University Hospital Okayama Japan; ^2^ Department of Paediatric Surgery Okayama University Hospital Okayama Japan; ^3^ Department of Gastroenterology and Hepatology Okayama University Hospital Okayama Japan; ^4^ Department of Haematology/Oncology Osaka Women's and Children's Hospital Osaka Japan

Acute graft versus host disease (GVHD) is a serious complication of allogenic haematopoietic cell transplantation. The standard treatment of acute GVHD is immunosuppressive drugs, but treating patients who do not respond to these drugs remains particularly challenging. Herein, we report a case of severe acute intestinal GVHD that caused multiple strictures of the small intestine, warranting surgical resection.

A 13‐year‐old boy with myelodysplastic syndrome underwent bone marrow transplantation (BMT) from an HLA‐matched unrelated donor. At 38 days post‐BMT, he presented with diarrhoea and abdominal pain, and acute intestinal GVHD was diagnosed.

Since his GVHD was steroid resistant, anti‐thymocyte globulin, methotrexate, and mesenchymal stromal cells were administered. His symptoms gradually improved over 6 months post‐BMT. However, 8 months post‐BMT, severe abdominal pain recurred. Multiple strictures of the ileum were observed on contrast‐enhanced X‐ray imaging and computed tomography (top); these strictures were assumed to be the fibrostenotic consequence of severe acute GVHD. These multiple strictures were so severe that passage of soft foods could not be expected. Ten months post‐BMT, he underwent temporary ileostomy to circumvent the strictures, and abdominal pain was ameliorated. Fifteen months post‐BMT, he underwent resection of the constricted ileum and ileostomy closure. Multiple strictures of the ileum were confirmed (bottom), and pathological inspection revealed fibrosis of the mucosa with no signs of persistent GVHD. The patient is now free from any gastrointestinal symptoms.

Severe acute intestinal GVHD induces strong inflammation and could lead to multiple severe fibrostenotic strictures of the intestine. Because acute GVHD is a systemic disease, surgery is not generally considered. The present case highlights the importance of recognising that a small subset of patients undergoing stem cell transplantation might develop severe intestinal GVHD warranting surgical intervention.

**FIGURE 1:**
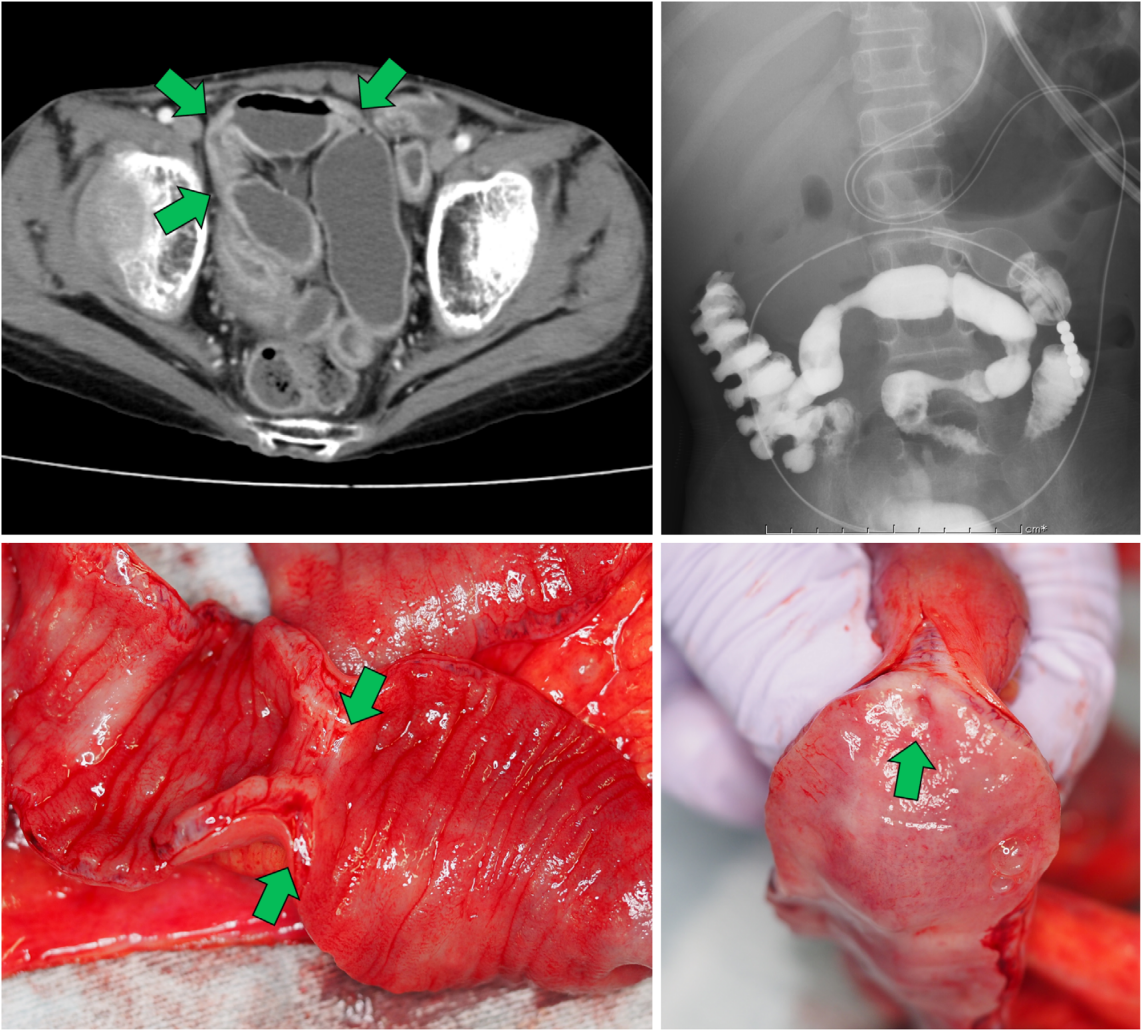
(top) Multiple strictures of the ileum were observed on contrast‐enhanced X‐ray imaging and computed tomography (bottom) Multiple strictures of the ileum were grossly confirmed at operation

## AUTHORS’ CONTRIBUTION

Hisashi Ishida wrote the manuscript. Hisashi Ishida, Takuo Noda, Seiji Kawano, Maho Sato, Hirokazu Tsukahara, and Akira Shimada participated in patient care. All authors reviewed the manuscript.

## CONFLICT OF INTEREST

The authors declare no conflict of interest.

## ETHICAL APPROVAL

Written informed consent was obtained from the patient and his mother.

## Data Availability

The data that support the findings of this study are available from the corresponding author upon reasonable request.

